# Tumor Microenvironment Acidosis and Alkalization-Oriented Interventions in Advanced Solid Tumors: A Narrative Review and Science-Based Medicine Perspective on Long-Tail Survival

**DOI:** 10.3390/cancers18081193

**Published:** 2026-04-08

**Authors:** Kazuyuki Suzuki, Shion Kachi, Hiromi Wada

**Affiliations:** 1 Department of Informatics, The University of Electro-Communications, 1-5-1 Chofugaoka, Chofu, Tokyo 182-8585, Japan; suzuki@uec.ac.jp; 2 Japanese Society on Inflammation and Metabolism in Cancer, 119 Nishioshikouji-cho, Nakagyo-ku, Kyoto 604-0842, Japan; aster_shion@kuhp.kyoto-u.ac.jp

**Keywords:** tumor microenvironment, acidosis, extracellular pH, immunotherapy, long-tail survival, science-based medicine

## Abstract

Median overall survival remains a central endpoint in oncology, but it can obscure a clinically meaningful long tail of patients with advanced solid tumors who survive well beyond the median. One biological context in which this pattern may be relevant is tumor microenvironment (TME) acidosis. Driven by aerobic glycolysis, hypoxia, impaired perfusion, and proton-export programs, acidic TME is increasingly implicated in invasion, therapeutic resistance, and immune suppression. This narrative review examines TME acidosis as the primary biological framework and considers long-tail survival as a clinical lens through which its implications may be interpreted. We summarize the biological basis and heterogeneity of acidic TME, review current approaches to clinical and translational assessment of tumor acidity, including acidoCEST magnetic resonance imaging (MRI) and positron emission tomography (PET)-based approaches, and discuss the potential and limitations of alkalization-oriented interventions such as buffering and diet-based strategies. Particular attention is given to the distinction between direct measurements of tumor acidity and clinically feasible but indirect markers such as urinary pH, which should not be interpreted as a direct surrogate for local tumor extracellular pH. From a science-based medicine perspective, long-tail survival is treated here as a hypothesis-generating clinical signal rather than proof of causality. Overall, alkalization-oriented interventions appear biologically plausible and clinically testable, but current clinical evidence remains limited and context-dependent. Future progress will require mechanistically informed biomarkers, careful safety evaluation, and trial designs capable of detecting delayed separation of survival curves and tail-oriented patterns of benefit.

## Simple Summary

Patients with advanced solid tumors are often judged mainly by median overall survival, but this number can miss a small group of patients who live much longer than expected. This review explains why the acidic environment around cancer cells may matter in that setting. Cancer cells often create acidity through abnormal metabolism, low oxygen, and poor blood flow. This acidic environment can help tumors invade surrounding tissue, resist treatment, and weaken anticancer immunity. We review approaches that may reduce this acidity, including buffering agents and diet-based strategies, while also discussing their limitations and the difficulty of measuring tumor acidity directly in patients. We also explain why urine pH should be viewed only as an indirect, supportive marker rather than a direct measure of acidity inside tumors. Overall, this review argues that targeting tumor acidity is biologically plausible, clinically testable, and potentially relevant to durable survival in selected patients.

## 1. Introduction

Despite major advances in systemic therapy, advanced solid tumors, especially stage IV disease, remain difficult to cure [1,2]. Within conventional evidence-based medicine (EBM), treatment evaluation still relies heavily on randomized controlled trials and population-average endpoints such as median overall survival (OS). Median OS remains indispensable for drug development, regulatory evaluation, and guideline formation. However, by definition, it does not fully describe the shape of the survival curve and cannot capture tail behavior beyond the point at which 50% of patients have died. In advanced cancer, survival curves often show a steep early decline followed by a more gradual tail, suggesting that a non-negligible subset of patients may experience prolonged disease control far beyond the median [3,4]. This long-tail pattern should not automatically be dismissed as a statistical curiosity. It may instead reflect clinically meaningful biological heterogeneity that is not adequately represented by median-based summaries alone.

One biological context in which this issue becomes particularly relevant is tumor microenvironment (TME) acidosis. Metabolic reprogramming in cancer, including aerobic glycolysis, together with hypoxia, impaired perfusion, and limited acid washout, promotes the production, retention, and export of lactate and protons, thereby lowering extracellular pH (pHe) [5,6,7,8,9]. Increasing evidence indicates that acidic TME is associated with invasion and metastasis, treatment resistance, and suppression of antitumor immunity [10,11,12,13,14,15]. At the same time, the extent, spatial distribution, and clinical significance of extracellular acidity are not uniform across tumor types, anatomical sites, disease stages, or treatment settings [16]. This heterogeneity complicates biological interpretation and clinical translation, but it also suggests that TME acidosis may represent a therapeutically relevant system-level feature rather than merely a byproduct of malignant metabolism.

In parallel, this review uses science-based medicine (SBM), as proposed by Wada and colleagues, as a conceptual framework [17,18]. Whereas current evidence-based medicine (EBM) is often applied in a predominantly deductive manner, starting from general premises and asking what should be observed if those premises are true, SBM also emphasizes the inductive significance of longitudinal patient-level observations that may not be fully explained within prevailing assumptions [17,18]. It is therefore introduced here not as a replacement for EBM or a rejection of population-based inference, but as a complementary and hypothesis-generating perspective that may be useful when conventional median-centered summaries do not fully capture clinically important patterns. From this viewpoint, cancer may be conceptualized as a dynamic tumor–host system integrating interacting processes involving metabolism, perfusion, inflammation, immunity, nutrition, and acid–base regulation. This systems-level perspective is informed by prior theoretical and methodological work on nonequilibrium systems, systems biology, and integrative cancer dynamics [19,20,21,22,23].

The primary aim of this narrative review is therefore not to reclassify survival statistics per se, but to examine TME acidosis and alkalization-oriented interventions as a biologically and clinically relevant framework in advanced solid tumors. Long-tail survival is discussed here as a clinical lens through which the implications of this framework may be interpreted, rather than as the sole or primary subject of the review. To this end, we first discuss the limitations of median OS in the interpretation of advanced cancer outcomes, then outline SBM as an integrative framework, review the biological basis and heterogeneity of TME acidosis, examine methods for assessing tumor acidity in patients, summarize current evidence and limitations regarding alkalization-oriented interventions, and finally consider implications for future trial design. In this manuscript, “alkalization” does not imply an attempt to raise blood pH directly. Rather, it refers to interventions intended to modify acid–base conditions relevant to the tumor–host environment, while acknowledging the uncertainty that remains between systemic markers and local tumor extracellular pH [24,25].

## 2. Limitations of Median Overall Survival in Advanced Cancer

Median overall survival (OS) is one of the most widely used outcome measures in oncology because it is simple, robust to skewed survival distributions, and clinically interpretable. However, it also has an inherent structural limitation: by definition, it is determined only by the time at which half of the study population has died and therefore does not describe the behavior of the remaining half of the survival curve beyond that point. In diseases in which a subgroup of patients experiences prolonged disease stabilization or unusually durable survival, median OS may underrepresent a clinically important aspect of benefit.

This limitation becomes even more relevant when treatment effects are delayed, heterogeneous, or concentrated in a subset of patients. In such circumstances, the hazard ratio may also become difficult to interpret, particularly when survival curves separate late. A therapy may meaningfully enrich the long-term survivor fraction while producing only a modest shift in the median, and the reverse may also occur. For this reason, median OS and hazard ratios should be regarded as necessary but not always sufficient descriptors of benefit in advanced cancer.

Several complementary approaches can help capture these patterns more adequately. Milestone survival rates at clinically meaningful time points, restricted mean survival time (RMST) within a pre-specified follow-up horizon, conditional survival probabilities, landmark analyses focused on longer-term survivors, and modeling approaches that allow for a cure-like or tail-enriched fraction may together provide a fuller description of clinical outcome. These approaches are not intended to replace conventional endpoints, but rather to supplement them when the biological context suggests that delayed or tail-oriented effects are plausible.

This consideration is especially relevant when the intervention of interest is hypothesized to act not primarily through immediate cytoreduction, but through gradual modification of the tumor–host environment. If TME-oriented interventions act by reducing immune suppression, altering metabolic stress, improving treatment tolerance, or weakening self-reinforcing malignant microenvironmental states, their clinical signature may emerge as delayed separation of survival curves or as an expanded long-term survivor fraction rather than as a large early shift in median OS. Accordingly, the statistical discussion in this review is included not as a separate methodological digression, but because the biological effects hypothesized for TME-oriented interventions may otherwise be underestimated when evaluated solely through median-based summaries.

## 3. Science-Based Medicine as a Framework

Science-based medicine (SBM), as used in this review, denotes a conceptual framework proposed by Wada and colleagues [17,18]. It begins with biologically grounded mechanisms, but it does not stop there. Rather, it seeks to connect mechanistic reasoning with longitudinal patient-level observations and clinical outcomes. In this sense, current evidence-based medicine (EBM) is often applied in a predominantly deductive manner: it starts from established general premises and asks what should be observed if those premises are true. SBM, by contrast, gives greater weight to the inductive significance of patient-level observations, especially when those observations are not fully captured by prevailing assumptions. It is therefore used here not to reject EBM, but to complement it by generating testable hypotheses in settings where clinical heterogeneity, delayed effects, and system-level interactions are biologically important.

From this perspective, cancer may be conceptualized not merely as an accumulation of molecular alterations, but as a dynamic tumor–host system integrating interacting processes involving metabolism, perfusion, stromal structure, immunity, inflammation, nutrition, and acid–base regulation. This systems-level perspective is informed by prior theoretical and methodological work on nonequilibrium systems, systems biology, and integrative cancer dynamics [19,20,21,22,23]. In this review, the term “attractor” is used heuristically to describe relatively stable, self-reinforcing operating modes of such a tumor–host system, and not as proof that clinical cancer trajectories have already been resolved into mathematically defined attractor states [26,27]. The value of this framework lies in its ability to organize heterogeneous observations into clinically relevant and ultimately falsifiable hypotheses.

TME acidosis represents one plausible example of such a system-level state [5,6]. Glycolytic metabolism, proton export, impaired perfusion, and reduced acid washout may reinforce one another and contribute to a lactate-rich, immune-suppressive, and therapy-resistant microenvironment [5,6,9,10]. One illustrative example is a tumor state in which high glycolytic flux, poor perfusion, and extracellular acidification reinforce one another, while on the host side impaired T-cell and natural killer (NK) cell effector function permits continued malignant progression [10,11]. A second illustrative example is a host state characterized by persistent systemic inflammation, poor nutritional status, limited treatment tolerance, and acid–base-related abnormalities, in which unfavorable clinical courses may cluster over time [17,28,29]. These examples do not establish discrete state transitions, but they help make the framework more concrete and clinically interpretable.

**Figure 1:**
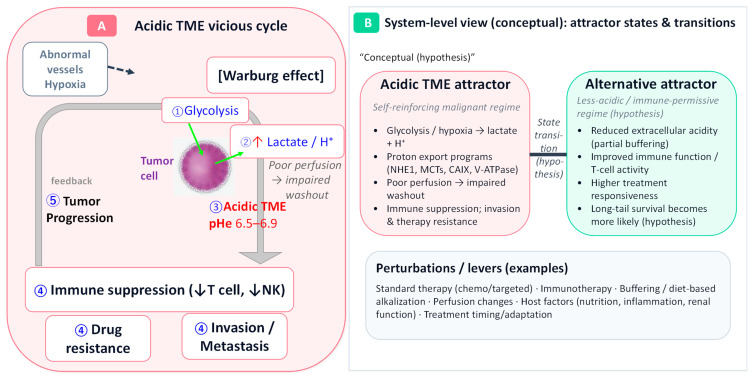
Acidic tumor microenvironment as a self-sustaining vicious cycle and a science-based medicine (SBM)-informed attractor-state model for long-tail survival. Panel (**A**) illustrates a vicious cycle in which enhanced glycolysis (the Warburg effect), together with lactate/proton export and acid retention caused by abnormal vasculature, poor perfusion, and hypoxia, promotes the formation and maintenance of an acidic tumor microenvironment (TME; extracellular pH (pHe) approximately 6.5–6.9). An acidic TME may in turn facilitate invasion/metastasis, drug resistance, and immunosuppression through impaired T-cell and NK-cell effector functions, thereby contributing to poor early outcomes in advanced cancer [5,6,7,8,9,10,13,14,15]. Panel (**B**) presents a conceptual schematic in which the tumor–host system is viewed as a non-equilibrium open system. Within this framework, an acidic TME is hypothesized to constitute a self-sustaining malignant regime (an attractor), whereas therapeutic interventions and/or changes in host conditions may help shift the system toward an alternative stable state characterized by lower acidity and more permissive immune function [19,20,21,22,23,26,27]. Arrows indicate proposed relationships and may include non-causal links; they should not be interpreted as evidence of established causality. Downward arrows (↓) indicate decreased effector function of immune cells, including T cells and natural killer (NK) cells. “Perturbations/levers” denote examples such as standard therapy, immunotherapy, buffering or dietary interventions, changes in perfusion, host factors (e.g., nutrition, inflammation, and renal function), and treatment timing or adaptive modifications [17,18,30,31,32,33,34,35,36,37,38].

Within this SBM framework, proximal markers can be treated as patient-level time series, allowing the hypothesized chain from intervention to acid–base environmental change, immune or metabolic change, and clinical outcome to be examined through testable predictions (Figure 1). If acidic TME functions as part of a self-reinforcing tumor–host state, then improvement in pH-related measures should tend to coincide with favorable changes in inflammatory, immune, or metabolic indicators rather than occur in isolation. Likewise, interventions that plausibly modify extracellular acidity may be more likely to produce delayed or tail-oriented benefit patterns than immediate universal tumor shrinkage. Conversely, if such coordinated longitudinal changes are not reproducible, or if no coherent relationship between pH-related markers and clinical outcome can be identified, then the usefulness of the model would be weakened. In this sense, SBM is used here as a hypothesis-generating and falsifiable interpretive framework for linking mechanism, longitudinal observation, and clinical heterogeneity, rather than as a claim of established causality.

## 4. Biological Basis of Tumor Microenvironment Acidosis

Tumor microenvironment (TME) acidosis should not be regarded as a passive or incidental byproduct of tumor metabolism. Rather, it is better understood as a biologically relevant condition that emerges through coordinated interactions among metabolic reprogramming, oxygen availability, vascular abnormalities, stromal architecture, and membrane transport systems [5,6,7,8,9,16]. Tumor cells generate lactate and protons through aerobic glycolysis while maintaining intracellular pH through transporters and buffering mechanisms such as the Na^+^/H^+^ exchanger, monocarboxylate transporters, carbonic anhydrase IX, and vacuolar ATPase [7,9,16]. In parallel, abnormal vasculature, hypoxia, and impaired perfusion limit the removal of acidic metabolites, thereby sustaining a relatively acidic extracellular environment [5,16].

As illustrated in Figure 1A, this acidic microenvironment may function as a self-reinforcing vicious cycle. Once established, it can promote multiple malignant phenotypes simultaneously. Acidic conditions favor local invasion and metastasis by promoting matrix remodeling, increasing cell motility, and selecting for phenotypes that tolerate environmental stress [12,13]. Acidosis may also contribute to therapeutic resistance by affecting the distribution and uptake of weakly basic drugs, activating adaptive stress programs, and interacting with hypoxia-related survival pathways [5,12]. In addition, accumulating evidence indicates that low extracellular pH and lactate accumulation can impair T-cell and NK-cell effector functions, reshape myeloid and stromal compartments, and contribute to an immunosuppressive TME [10,11,14,15]. In this sense, acidic TME can be viewed as a biologically convergent node linking invasion, resistance, and immune dysfunction.

Importantly, however, acidic TME is not a uniform feature of all cancers to the same degree [16]. The extent, spatial distribution, and clinical implications of extracellular acidosis vary according to tumor type, anatomical site, disease stage, vascularity, perfusion status, stromal composition, hypoxic burden, and treatment history [16,39]. Even within a single lesion, extracellular pH may be spatially heterogeneous, with more severe acidification in poorly perfused or highly glycolytic regions [5,16]. This context dependence is clinically important because the same pH-oriented intervention may have different biological leverage across tumor settings and therefore should not be discussed as though it would exert a universal effect in all cancers.

Recent preclinical and animal studies further support the view that acidosis is not merely descriptive but can act as a selective microenvironmental condition that shapes tumor behavior [13,14,15]. At the same time, these data suggest that acidity does not operate as an isolated variable. Rather, its effects are intertwined with hypoxia, nutrient stress, stromal biology, and host immunity [10,12,39]. Accordingly, TME acidosis is best understood as an integrative and context-dependent biological condition whose significance depends on tumor type and disease stage, host state, and treatment setting. This interpretation provides the biological basis for the conceptual model shown in Figure 1B and also supports the rationale for considering alkalization-oriented interventions as one possible strategy for perturbing maladaptive tumor–host dynamics.

## 5. Clinical and Translational Assessment of Tumor Acidity

Direct assessment of tumor acidity in patients remains technically challenging. Unlike many intracellular molecular events that can be studied using tissue assays, extracellular tumor pH varies across space and time and is not easily captured through routine clinical measurement [24,30,40,41,42]. Current approaches therefore range from imaging-based techniques that attempt to evaluate pH in vivo to indirect biochemical or physiological markers that reflect related systemic processes [24,30,40,41,42]. These methods do not measure the same phenomenon, and each has distinct strengths and limitations (Table 1).

Among the more direct or near-direct methods, magnetic resonance-based techniques are of particular interest. Chemical exchange saturation transfer magnetic resonance imaging (MRI), including acidoCEST MRI, has been developed to estimate extracellular tumor pH with spatial resolution and has shown promise in preclinical and early translational settings [40]. Hyperpolarized ^13^C-bicarbonate MRI can also provide dynamic information relevant to tissue acid–base chemistry and has expanded interest in noninvasive pH-related imaging [41]. These techniques are attractive because they may allow spatial mapping of pH heterogeneity and assessment of changes after metabolic or buffering interventions. However, wider clinical adoption remains limited by technical complexity, acquisition time, specialized infrastructure, and limited standardization across centers [40,41].

Positron emission tomography (PET)-based approaches are also relevant, but their interpretation requires precision. pH-sensitive PET tracers and related investigational probes may provide more direct information about acidic tumor regions, although many remain at an early translational stage [24]. By contrast, conventional ^18^F-fluorodeoxyglucose (FDG) positron emission tomography (PET) does not directly measure extracellular tumor pH [42]. Rather, it reflects glucose avidity and, in many tumor contexts, metabolic activity associated with enhanced glycolysis that may favor lactate and proton production [7,8,42]. In addition, lactic acidosis within the tumor microenvironment may itself influence intratumoral heterogeneity of ^18^F-FDG uptake, further underscoring that FDG PET should be interpreted as an indirect metabolic readout rather than a direct measure of extracellular tumor pH [42]. Accordingly, ^18^F-FDG PET should be regarded as an indirect metabolic correlate of acid-producing tumor states, not as a surrogate measure of tumor acidity itself.

In addition to imaging, several biochemical and physiological measures have been considered as indirect markers related to tumor acidity [24,30,42]. Circulating metabolites, lactate-related indices, and systemic acid–base parameters may provide partial insight into the metabolic context of the host, but they do not specifically represent local tumor pH [24,30,42]. From a practical clinical perspective, urinary pH has attracted attention because it can be measured repeatedly and noninvasively over time [18,25,31,44]. However, urinary pH reflects renal acid excretion and systemic acid–base regulation rather than local tumor extracellular pH and therefore should not be interpreted as a direct surrogate for tumor acidity [25]. Its possible value lies instead in its role as a supportive longitudinal marker and, in some settings, as a clinically feasible indicator related to adherence or acid load, provided that it is interpreted cautiously and together with other biological and clinical data [18,31].

Taken together, the translational challenge is not to force all markers into a single hierarchy, but to interpret them according to what they measure. Imaging-based methods, indirect metabolic correlates, and longitudinal systemic markers each provide different information and should ideally be combined within mechanistically informed study designs [24,30,40,41,42]. Future progress will likely depend on multimodal strategies that integrate spatial imaging, metabolic context, and longitudinal clinical data, allowing tumor acidity to be characterized more plausibly and rigorously in human studies. In this sense, direct pH-oriented imaging, indirect metabolic correlates, and longitudinal systemic markers should not be treated as interchangeable, but rather as complementary components of a coherent translational framework.

Despite these measurement limitations, the biological and preclinical rationale is sufficient to support cautious translational consideration of alkalization-oriented interventions.

## 6. Alkalization-Oriented Interventions

If acidic TME contributes to invasion, therapeutic resistance, and immune dysfunction, then interventions that attenuate tumor acidity warrant consideration as adjunctive therapeutic strategies [14,15,32,33]. Having outlined above how tumor acidity may be assessed and the limitations of current biomarkers, the key translational question here is not whether alkalization alone can universally control advanced cancer, but whether selected interventions can favorably modify the biological context in which standard therapies act. In this review, alkalization-oriented interventions are therefore discussed as candidate modifiers of tumor–host conditions rather than as established stand-alone anticancer therapies.

Operationally, these interventions may be grouped into three broad categories. The first includes buffering-oriented approaches intended to alter systemic or local acid–base conditions, such as oral bicarbonate- or citrate-based strategies [34,35,36]. The second includes diet-based approaches aimed at reducing dietary acid load or modifying metabolic context in a manner that may influence systemic buffering and related physiological conditions [25,45]. The third includes combination-oriented approaches in which alkalization-related measures are used alongside chemotherapy, immunotherapy, or other standard treatments in the expectation that modifying microenvironmental stress may enhance overall therapeutic effectiveness [33,35,36,37,38]. In addition, the broader pH-targeted literature, including approaches directed at proton export and carbonic anhydrase activity, provides contextual evidence that tumor acidity is pharmacologically and biologically tractable, even when those strategies fall outside the narrower operational definition of “alkalization” used here [12,32,33,43]. In this context, recent review literature also emphasizes that pH regulation in acidic TME is closely linked to its immunological state, further supporting interest in alkalization-oriented and related pH-targeted interventions [43].

At present, the strongest support for alkalization-oriented interventions comes from preclinical and proof-of-concept studies, including animal experiments suggesting that buffering or pH-targeted manipulation can influence tumor invasion, metastatic behavior, or treatment response in selected contexts [15,32,34,35,36]. However, translation to human clinical practice remains limited [37,38,45]. Clinical observations reported to date, including those involving urinary pH and outcome, are of interest but remain observational, heterogeneous, and insufficient to establish a generalizable conclusion regarding efficacy [18,25,31,37,38]. Accordingly, the current literature supports biological plausibility and clinical testability, but not definitive efficacy.

Several uncertainties remain central to this field. Local tumor pH is heterogeneous and difficult to measure directly in routine practice [24,30,40,41,42]. Biomarker validation remains incomplete, and clinically accessible markers such as urinary pH are indirect rather than mechanistically specific [25]. Adherence to dietary interventions can vary substantially, buffering agents may impose sodium or electrolyte burdens, renal function and comorbidity may influence both safety and interpretation, and it is often difficult to confirm whether a given intervention has modified tumor acidity in humans [25,35,36,45]. These challenges do not invalidate the concept, but they do mean that claims should remain cautious and explicitly context-dependent.

Taken together, alkalization-oriented interventions remain exploratory, although they appear biologically plausible and clinically testable. Their potential value may lie less in direct tumoricidal action than in altering the tumor–host environment so that standard therapies can operate under more favorable biological conditions. Future work should therefore prioritize context-specific patient selection, integration with standard systemic therapy, mechanistically informed biomarker strategies, and rigorous prospective evaluation.

## 7. Long-Tail Survival as a Clinical Perspective

In this review, long-tail survival is introduced not as an independent statistical topic, but as a clinical perspective through which the biological and translational relevance of tumor microenvironment (TME) acidosis may be interpreted. The purpose of this section is to consider whether durable survival patterns observed in a subset of patients may reflect processes related to tumor acidity, host adaptation, and the modifying effects of alkalization-oriented interventions. This perspective is not intended to imply proven efficacy of alkalization-oriented interventions, but rather to examine whether durable survival patterns may help generate mechanistically informed hypotheses.

Long-term survival in advanced cancer is not always characterized by an early dramatic tumor response. In some patients, durable disease stabilization appears to emerge gradually over time, even in the absence of radiographic complete response. In such cases, the tumor may enter a state compatible with prolonged coexistence with the host while preserving functional status and quality of life [17,28,29]. This clinical pattern suggests that long-term outcomes may not be determined solely by the magnitude of early tumor shrinkage, but also by broader biological conditions involving the TME, host immunity, metabolism, and systemic resilience.

From this perspective, long-tail survival can be considered a clinically relevant pattern that may reflect underlying biological heterogeneity rather than a purely statistical anomaly. Within the science-based medicine (SBM) framework, one hypothesis is that a subset of patients may experience a shift in the tumor–host system from a more aggressive state, characterized by glycolytic dominance, extracellular acidosis, and immune dysfunction, toward a more stable state associated with improved host–tumor balance and more durable disease control. This interpretation should not be viewed as established proof of a discrete state transition, but rather as a hypothesis-generating framework for integrating mechanistic and longitudinal clinical observations. Accordingly, the SBM-based interpretation proposed here is intended primarily as a heuristic framework for organizing observations and generating testable hypotheses. Importantly, the occurrence of long-tail survival does not in itself establish a specific biological mechanism. Instead, it highlights the possibility that heterogeneous tumor–host interactions, including microenvironmental factors such as acidity, may influence long-term outcomes in selected patients.

As an illustrative example, Kaplan–Meier curves reported in patients with stage IV pancreatic cancer and stratified by urinary pH show an early truncation of survival in the low-urinary pH group, whereas a longer survival tail persists in patients with higher-urinary pH values [31]. This pattern is visually suggestive and may be clinically relevant in the context of an acid–base-related intervention. However, the observation should be interpreted cautiously because the analysis is observational, subgroup sizes are limited, and residual confounding cannot be excluded.

In the underlying analysis, representative urinary pH was not defined from a single time point but from repeated measurements [31]. For each patient, representative urinary pH was defined as the mean of all available urinary pH measurements obtained on at least three separate occasions. Urinary pH was assessed repeatedly during the observation period from January 2014 through 30 April 2024. Based on the extracted time-series dataset, the median number of urinary pH measurements per patient was 4, with an interquartile range of 2 to 9 measurements, and the median interval between consecutive measurements was 27 days, with an interquartile range of 15 to 35 days. This approach was adopted to reduce the influence of short-term fluctuation and to provide a more stable indicator of systemic acid–base status over time. Nevertheless, urinary pH should not be interpreted as a direct surrogate for local tumor extracellular pH, because it primarily reflects renal acid excretion and systemic acid–base regulation rather than tumor pHe itself.

To address potential confounding by baseline characteristics, we additionally examined a Cox proportional hazards model including age at first visit, sex, body weight, and urinary pH [31]. In this multivariable analysis, urinary pH remained significantly associated with overall survival (*p* = 0.0074), whereas age, sex, and body weight were not statistically significant. These findings suggest that the association between urinary pH and survival was not fully explained by these baseline covariates alone. This multivariable result should therefore be interpreted as supportive of a persistent association within the limits of the available dataset, rather than as evidence of an independent causal effect of urinary pH on survival. However, this result should still be interpreted cautiously because the sample size was limited, subgroup imbalance remained possible, and unmeasured confounding factors, including disease burden, treatment intensity, nutritional status, renal function, and systemic metabolic state, may have influenced the observed association.

**Figure 2:**
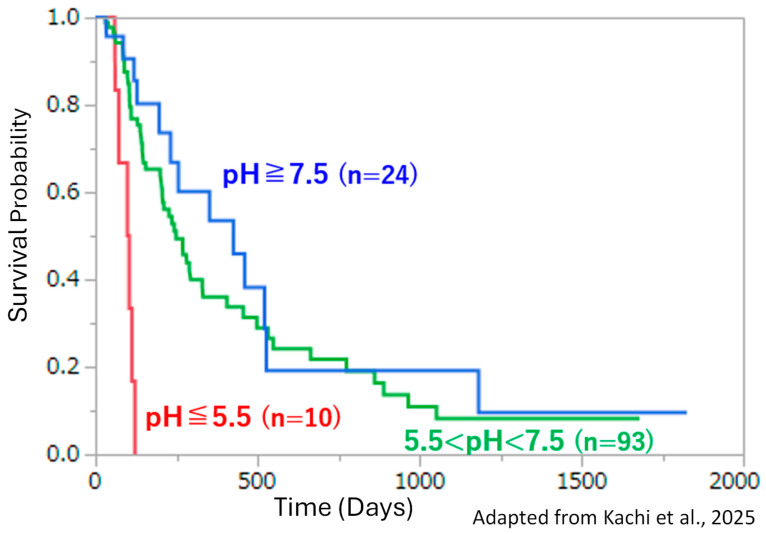
Kaplan–Meier survival curves stratified by representative urinary pH in stage IV pancreatic cancer (Kachi et al., 2025) [31]. Patients were stratified into three groups according to representative urinary pH (Group 1: pH ≥ 7.5, n = 24; Group 2: 5.5 < pH < 7.5, n = 93; Group 3: pH ≤ 5.5, n = 10). Representative urinary pH was defined for each patient as the mean of all available urinary pH measurements obtained on at least three separate occasions, in order to reduce the influence of fluctuation associated with single measurements. Urinary pH was assessed repeatedly during the observation period from January 2014 through 30 April 2024. Based on the extracted time-series dataset, the median number of urinary pH measurements per patient was 4, with an interquartile range of 2 to 9 measurements, and the median interval between consecutive measurements was 27 days, with an interquartile range of 15 to 35 days. Differences among groups were significant by the log-rank test (χ^2^ = 22.6, df = 2, *p* < 0.0001) and also by the Wilcoxon test (χ^2^ = 17.5, df = 2, *p* = 0.0002). Median survival times were 426.0 days (Group 1), 249.0 days (Group 2), and 101.5 days (Group 3). Five-year survival rates were 9.6% (Group 1), 8.1% (Group 2), and 0% (Group 3). In an exploratory Cox proportional hazards model including age at first visit, sex, body weight, and urinary pH, urinary pH remained significantly associated with overall survival (*p* = 0.0074), whereas age, sex, and body weight were not statistically significant. Because this analysis was observational and subgroup sizes were limited, the findings should be interpreted as hypothesis-generating rather than definitive evidence of causality. This figure was prepared and modified based on Kachi et al. [31]. Adapted with permission from Ref. [31]. © 2025 the author(s), published by De Gruyter on behalf of Tech Science Press (TSP); licensed under CC BY 4.0.

Accordingly, urinary pH should not be interpreted as a direct surrogate for tumor extracellular acidity, nor should the survival differences shown in Figure 2 be regarded as definitive evidence that modulation of tumor acidity caused the long-tail pattern. Rather, these findings are best viewed as hypothesis-generating clinical observations that justify further mechanistically informed investigation. In this context, repeated urinary pH measurements may still be informative when treated as longitudinal pH-related markers and interpreted together with clinical trajectories and other biological indicators.

A more rigorous evaluation of this hypothesis will require integration of urinary pH with longitudinal data on inflammation, immune status, metabolic variables, treatment exposure, and disease burden [17,18,29,44]. Rather than relying on a single pH measurement, future studies should model urinary pH as a time-varying trajectory and examine whether changes in this trajectory are associated with delayed separation of survival curves, durable disease control, or changes in other mechanistically relevant biomarkers. Analytical approaches such as time-varying covariate models, robust summary measures, landmark analyses, and tail-focused survival metrics may be useful in this regard.

Importantly, the absence of a simple one-to-one relationship between urinary pH and tumor pHe does not invalidate the broader conceptual model. Rather, it underscores the need to distinguish between direct mechanistic measurements and clinically feasible longitudinal markers. If pH-related interventions are found to correlate with favorable immune, inflammatory, or metabolic changes over time, and if these associations coincide with more durable survival patterns, the hypothesis that modulation of the tumor–host environment contributes to long-tail survival would gain support. Conversely, if such associations are not reproducible, or if no coherent biological correlates can be identified, the proposed interpretation would need to be reconsidered.

Thus, long-tail survival is best regarded not as proof of a specific mechanism, but as a clinically meaningful survival pattern that may reflect important biological processes not fully captured by median overall survival alone. In this review, it serves as a clinical perspective through which the relevance of TME acidosis and alkalization-oriented interventions may be interpreted, while highlighting the need for more rigorous longitudinal and mechanistically grounded validation in future studies.

## 8. Implications for Future Clinical Trial Design

If TME acidosis is biologically relevant and alkalization-oriented interventions are to be evaluated credibly, future clinical trials must be designed to assess both mechanism and outcome. A convincing development pathway in this field should therefore pre-specify not only conventional clinical endpoints, but also biomarkers and analytical strategies capable of detecting delayed, heterogeneous, and tail-oriented patterns of benefit. This is especially important because interventions that modify the tumor microenvironment may not produce immediate universal tumor shrinkage even when they have biologically meaningful effects.

Mechanistic endpoints may include direct or near-direct imaging-based assessments of tumor acidity, such as acidoCEST MRI where feasible, together with lactate-related measures, inflammatory indices, immune readouts, and carefully interpreted longitudinal pH-related markers. Clinical endpoints should extend beyond median OS and hazard ratios to include milestone survival, restricted mean survival time within a pre-specified horizon, conditional survival focused on longer-term survivors, and appropriately planned landmark analyses. In selected settings, modeling approaches that allow for a cure-like or tail-enriched fraction may also be informative. The purpose of this broader endpoint strategy is not to inflate significance, but to align trial design more closely with plausible biology.

Patient selection and stratification are also likely to be crucial. In addition to tumor type, stage, histology, and molecular features, future studies may need to consider baseline metabolic state, inflammatory condition, renal function, treatment exposure, and the feasibility of repeated pH-related measurements. Because acidic TME is context-dependent, it is unlikely that a single trial design will fit all tumor settings equally well. Enrichment strategies based on biological plausibility may therefore be more informative than assuming universal applicability.

Within this framework, urinary pH may have a role, but only within its appropriate limits. It should not be used as a one-time baseline surrogate for tumor extracellular pH. Rather, if included, it should be analyzed longitudinally as a time-varying or trajectory-based marker that may reflect acid–base-related clinical context, adherence, or systemic physiological shifts over time. More direct measures of tumor acidity, when feasible, should be integrated with longitudinal immune, inflammatory, and metabolic data so that observed clinical patterns can be interpreted within a more plausible mechanistic chain.

Because alkalization-oriented interventions are unlikely to function as universal monotherapy, combination-oriented designs with adequate follow-up duration are especially important. Trials should be sufficiently long to detect delayed separation of survival curves, and analyses should be planned prospectively to avoid reducing potentially meaningful long-tail patterns to secondary or post hoc observations. The ultimate goal is not merely to show statistical difference, but to determine whether a pH-oriented intervention plausibly alters the biological pathway linking TME acidity, host response, and long-term clinical outcome.

## 9. Conclusions

TME acidosis provides a biologically coherent but context-dependent framework for understanding how metabolism, perfusion, immune suppression, and therapeutic resistance may interact in advanced solid tumors. Its importance lies not in a claim of universality, but in the possibility that extracellular acidification functions as an integrative feature of tumor–host biology whose relevance varies across tumor settings.

Within this perspective, alkalization-oriented interventions should be regarded, at present, as mechanistically plausible but incompletely validated strategies. Their clinical relevance remains to be established through better biomarkers, context-specific patient selection, careful safety evaluation, and more rigorous prospective testing. In particular, future progress will depend on improving the distinction between direct assessments of tumor acidity and indirect but clinically feasible longitudinal markers.

Long-tail survival is best interpreted here not as proof of a specific mechanism, but as a clinically meaningful pattern that may reveal biological processes not fully captured by median-based outcomes alone. From the perspective adopted in this review, such patterns are valuable because they can generate mechanistically informed hypotheses regarding tumor acidity, host adaptation, and durable disease control, while still requiring careful empirical validation.

Whether modulation of acidic TME can reproducibly contribute to prolonged survival remains an open question. However, this question is biologically grounded, clinically relevant, and testable. Clarifying this issue may improve not only the development of pH-oriented strategies, but also the broader interpretation of durable survival patterns in advanced cancer beyond the limitations of conventional summary metrics.

## Figures and Tables

**Table 1:** Clinical and Translational Methods for Assessing Tumor Acidity.

Modality/Marker	What it Primarily Measures	Strengths	Main Limitations	Current Translational Stage	Most Appropriate Role in Clinical Studies
acidoCEST MRI [32,40]	Spatially resolved extracellular tumor pH (pHe) using chemical exchange saturation transfer contrast.	One of the more direct noninvasive approaches for mapping pHe heterogeneity; suitable for pharmacodynamic assessment in mechanism-focused studies.	Requires dedicated sequences, contrast agents, technical expertise, and cross-center standardization; availability remains limited.	Preclinical to early clinical translation.	Pharmacodynamic imaging biomarker in mechanism-oriented trials and translational pilot studies.
Hyperpolarized ^13^C-bicarbonate MRI/related hyperpolarized pH imaging [41]	Dynamic tissue acid-base chemistry and pHe-related information.	Highly mechanistically informative; may be useful for early response assessment in specialized research settings.	Requires specialized hardware, rapid workflow, tracer preparation, and high technical support; currently limited to highly specialized centers.	Preclinical/early translational.	Research biomarker in specialized translational studies.
pH-sensitive PET tracers/PET-MRI co-agents [24]	Acidity-oriented tracer uptake or pH-responsive signal.	Potentially compatible with whole-body imaging and clinical nuclear medicine workflows.	Many tracers remain investigational; specificity, in vivo stability, and regulatory availability are still limited.	Investigational/early translational.	Exploratory imaging endpoint when whole-body assessment of acidity is desired.
Conventional ^18^F-FDG PET [42]	Glucose avidity and glycolysis-associated metabolism, not extracellular tumor pH itself.	Widely available; useful for metabolic context, lesion selection, and assessment of tumor heterogeneity.	Indirect only; FDG uptake is influenced by factors other than acidity, including proliferation, inflammation, perfusion, and lactic acidosis.	Routine clinical use, but only indirect for acidity-related interpretation.	Contextual metabolic correlate to be interpreted alongside pH-directed imaging or other mechanistic biomarkers.
Urinary pH	Renal acid excretion and systemic acid-base handling rather than local tumor pHe.	Simple, inexpensive, repeatable, and feasible for longitudinal monitoring in routine practice.	Strongly influenced by diet, hydration, renal function, medications, and sampling timing; not a direct tumor biomarker.	Routine clinical test; exploratory in oncology-specific pH research.	Supportive longitudinal marker and possible adherence/acid-load indicator, not a surrogate endpoint for tumor acidity.
Circulating lactate, serum bicarbonate, and other systemic acid-base measures [30,43]	Systemic metabolic and acid-base context.	Clinically accessible; may complement imaging and help characterize host physiological context.	Low tumor specificity and limited ability to localize or quantify intratumoral pHe heterogeneity.	Routine laboratory measures with exploratory translational use.	Adjunct host-context markers in multimodal biomarker panels.

Notes: Conventional ^18^F-FDG PET should be interpreted as an indirect metabolic correlate of acid-producing tumor states and not as a direct measure of tumor extracellular pH. Urinary pH reflects systemic and renal acid-base handling and should be treated as a supportive longitudinal marker rather than a direct surrogate of local tumor acidity. The “Main Limitations” listed in this table are based on the cited references. Abbreviations: pHe, extracellular pH; CEST, chemical exchange saturation transfer; PET, positron emission tomography; FDG, fluorodeoxyglucose.

## Data Availability

No new data were created or analyzed in this study. Data sharing is not applicable.
